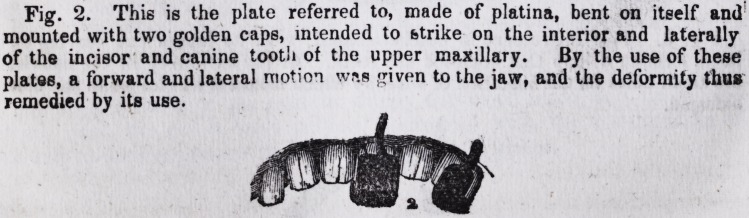# Case of Irregular Arrangement, and the Remedy

**Published:** 1840

**Authors:** B. A. Rodriguez


### MEMORANDA.

*From the Note Book of B. A. Rodriguez, M. D.*

Elizabeth, daughter of Mr. Klink, called with her father to consult on the practicability of relieving a deformity of the lower maxillary bone. From his earliest recollections, his daughter had an obvious projection of this bone forward, and laterally to the right, disfiguring her face (Fig. 1) and impairing somewhat her utterance. So complete was the protrusion, that the upper row of teeth struck fully an eighth of an inch to the anterior of the lower; and the first incisor on the right, fell behind the first lower incisor of the same side, presenting a deformity that rendered the patient and her parents uneasy, and resolved to hazard all attempts that promised success, to rectify it. After close scrutiny, I saw nothing in the mobility of the joint, the absence of malformation, and the slight inconvenience a twist of the bone, to its proper position gave, that did not warrant fully, any attempt to repair the disorder. In truth, I regarded it more as deformity of habit, than a natural one, by which the lateral motion of the joint was more exercised, and the muscles of the right side disposed, from long use, to retain the bone in that position. I thought, if by any means, I could restore the bone to its nataral position, and retain it there a sufficient length of time, till the muscles of the left side, viz: the masseter and pterygoid, which preside over the lateral and forward motions of the joint, had regained their power of contractility, success must follow.

Regarding it more as the result of the loss of such power in these muscles than any lesion of the joint, I invented the metallic plates, which will be found in (Fig. 3.) and which, after the patient had worn for one month, fully answered the purpose intended. The lateral deformity is radically obviated, as the engraving, illustrating the original defect and

---

Fig. 1. This was the appearance of the child when she called on me for assistance. The projection is manifest, both to the right and forward, and the deformity such as to disfigure and incommode her.

Fig. 1.

Fig. 3. This instrument is hatchet-shaped, with its longer end curving on itself, forming a loop that enclosed the tooth. At the extremity of the handle, are small holes for the insertion of wire, by which means it was lashed to the first bicuspid.

the remedy will shew. But here but one half of the undertaking was accomplished. After restoring the jaw to its natural form, I found that the upper row of teeth still struck on the interior of the lower, and that whenever the patient separated her lips the same ugly lapping of the lower jaw over the upper was visible. The mouth and teeth presented the reverse appearance, uniformly met with, and resembled strongly the sullen grin of the bull dog. To obviate this, I invented the accompanying instrument, (Fig. 2.) so arranged when placed on the upper central incisor of the right, and secured to the first bicuspid, that it operated as a lever in giving an outward direction to the tooth, and restoring it to its proper and natural condition. The constant use of this instrument has so far and fully rectified the second difficulty, that she now articulates without restraint; enjoys a free use of the jaw and differs in no particular from the ordinary appearance of other persons. Her parents are satisfied and she herself rejoices that Art has been able to triumph so signally over the freaks of Nature.

---

*For the American Journal of Dental Science.*

Fig. 2. This is the plate referred to, made of platina, bent on itself and mounted with two golden caps, intended to strike on the interior and laterally of the incisor and canine tooth of the upper maxillary. By the use of these plates, a forward and lateral motion was given to the jaw, and the deformity thus remedied by its use.

## Figures and Tables

**Fig. 1. f1:**
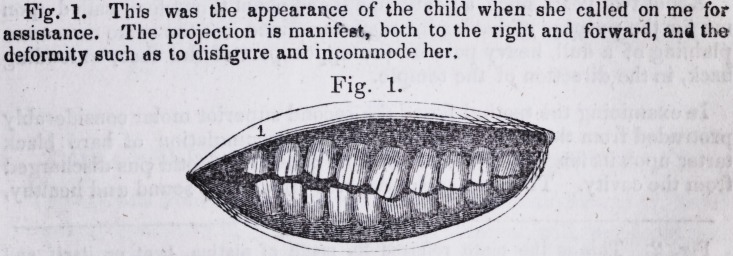


**Fig. 3. f2:**
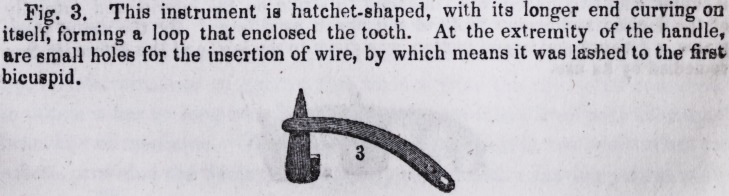


**Fig. 2. f3:**